# Estimation of the Population Size of Men Who Have Sex With Men in Vietnam: Social App Multiplier Method

**DOI:** 10.2196/12451

**Published:** 2019-04-17

**Authors:** Vo Hai Son, Ali Safarnejad, Nguyen Thien Nga, Vu Manh Linh, Le Thi Cam Tu, Pham Duc Manh, Nguyen Hoang Long, Abu Abdul-Quader

**Affiliations:** 1 Vietnam Authority of HIV/AIDS Control Ministry of Health Hanoi Vietnam; 2 The Joint United Nations Programme on HIV/AIDS Green One United Nations House Hanoi Vietnam; 3 Centers for Disease Control and Prevention Hanoi Vietnam

**Keywords:** HIV, AIDS, population size estimation, men who have sex with men, respondent-driven sampling, Vietnam

## Abstract

**Background:**

Although the prevalence of HIV among men who have sex with men (MSM) in Vietnam has been increasing in recent years, there are no estimates of the population size of MSM based on tested empirical methods.

**Objective:**

This study aimed to estimate the size of the MSM population in 12 provinces in Vietnam and extrapolate from those areas to generate a national population estimate of MSM. A secondary aim of this study was to compare the feasibility of obtaining the number of users of a mobile social (chat and dating) app for MSM using 3 different approaches.

**Methods:**

This study used the social app multiplier method to estimate the size of MSM populations in 12 provinces using the count of users on a social app popular with MSM in Vietnam as the first data source and a questionnaire propagated through the MSM community using respondent-driven sampling as the second data source. A national estimation of the MSM population is extrapolated from the results in the study provinces, and the percentage of MSM reachable through online social networks is clarified.

**Results:**

The highest MSM population size among the 12 provinces is estimated in Hanoi and the lowest is estimated in Binh Dinh. On average, 37% of MSM in the provinces surveyed had used the social app Jack’d in the last 30 days (95% CI 27-48). Extrapolation of the results from the study provinces with reliable estimations results in an estimated national population of 178,000 MSM (95% CI 122,000-512,000) aged 15 to 49 years in Vietnam. The percentage of MSM among adult males aged 15 to 49 years in Vietnam is 0.68% (95% CI 0.46-1.95).

**Conclusions:**

This study is the first attempt to empirically estimate the population of MSM in Vietnam and highlights the feasibility of reaching a large proportion of MSM through a social app. The estimation reported in this study is within the bounds suggested by the Joint United Nations Programme on HIV/AIDS. This study provides valuable information on MSM population sizes in provinces where reliable estimates were obtained, which they can begin to work with in program planning and resource allocation.

## Introduction

### Background

Since the first reported case of HIV in Vietnam in 1990 [1-3], over 400,000 people have been estimated to be infected with HIV in the country and 150,000 are reported to have died of AIDS-related causes [4]. The HIV epidemic in Vietnam is concentrated among 3 key populations (ie, people who inject drugs, men who have sex with men [MSM], and female sex workers) and their sexual partners [5]. The main route of transmission has been through sharing of needles when injecting drugs, followed by sexual transmission; however, the proportion attributable to the latter mode of transmission has been increasing in recent years [4]. By 2015, the estimated number of new infections had decreased by 50% from the peak of the epidemic in 2002, thanks to prevention initiatives for key populations, including provision of clean needles and syringes, condoms, methadone maintenance therapy, and antiretroviral treatment [4,6].

Although the overall number of new infections has declined, the prevalence of HIV among MSM has been increasing in recent years [4]. HIV sentinel surveillance data for MSM in 2014 in 8 provinces found an average HIV prevalence of 6.7%, with higher prevalence reported in major urban areas, and 2017 surveillance in 9 provinces found an average HIV prevalence of 12.2% [7,8]. Despite these alarming estimates, information about the population size of the MSM community is limited and imprecise.

Estimates of the size of populations at risk of HIV are necessary to understand the scale of the epidemic and in planning appropriate interventions and allocation of resources. A number of estimates of the MSM population size have been attempted in Vietnam, targeted to limited provinces [9]. The national estimate of the MSM population size in Vietnam has not been based on any empirical method of estimation. A frequently used method to approximate the range of the MSM population is to take a percentage of the male population as being MSM, based on regional percentages and consensus among experts, and adjust it by the level of urbanization [10-12]. In Vietnam, the accepted MSM population size is concluded on by the national technical working group, which updates the epidemiological model for the country and revises the expected MSM population size, among other input values to the model, until it produces a validated epidemiological prediction. The MSM population size based on this modeling process in Vietnam was around 330,000 in 2016.

### Objectives

Besides the inherent imprecision of the methods to arrive at the MSM population size based on profiling of urbanization of regions and modeling, the previous accepted population size of MSM in Vietnam does not reveal much about how this population can be reached, how MSM network together, what regional variations exist, or the age demographics of the reachable MSM. This study attempted to directly estimate the size of the MSM population in 12 provinces in Vietnam using the social app multiplier method and extrapolate from those areas to generate a national population estimate of MSM. A secondary aim of this study was to compare the feasibility of obtaining the number of users on a social app for MSM using 3 different approaches.

## Methods

### Social App Multiplier Overview

This study used the social app multiplier method to estimate the population size of MSM in 12 provinces of Vietnam. This method was piloted in Ho Chi Minh City and Nghe An province in 2016 and subsequently updated and improved based on learnings from the pilot study [9]. In the formative phase of this study, 12 out of 63 provinces of Vietnam were selected that were predicted to have the greatest size of MSM population, which represent a diversity of the regions of the country, and where there exist a minimum number of social app users so that the method could be applied. These 12 provinces were An Giang, Bac Giang, Binh Dinh, Can Tho, Da Nang, Dak Lak, Dong Nai, Dong Thap, Hanoi, Hai Phong, Nam Dinh, and Thanh Hoa. The next sections briefly describe the methods used in this study. They follow the general multiplier method, which compares 2 independent data sources to estimate the total number in a population, the first source being a count from program data that include only the population whose size is being estimated and a second source being a representative survey of the population whose size is being estimated [13]. Additional details of the data collection procedures have been previously described in the pilot study [9].

### Count of Social Apps’ Users

The social app Jack’d was selected to provide the first count. Overall, 3 methods were used to count the total number of users on Jack’d. First, following the method used during the pilot, in each of the 12 provinces, the total number of active users on Jack’d was enumerated over a 1-month period, and the final list of active users was deduplicated using the public profile information of app users such as age, pseudonym, and avatar. When counting active users, only profiles that appeared at least twice and spaced by several days in between were included in the final count to minimize the possibility of counting short-term visitors. A second method was a capture-recapture procedure that matched active users on Jack’d at 2 different time points, using the same public profile information as used in the first method, to estimate the total number of active users on the app in each of the respective provinces. The third method was to procure the total aggregate, unduplicated, and nonidentifiable number of app users in the respective province over a period of 1 month directly from the Jack’d social app administrator.

### Respondent-Driven Sampling Online Survey

Immediately after 1 month of counting users on the Jack’d social app, an online survey using respondent-driven sampling (RDS) recruitment strategy was conducted in the MSM community in each of the 12 provinces to find out about their use of Jack’d in the past month. RDS is a form of chain referral sampling that uses a mathematical model to approach a true random sample [14]. The inclusion criteria for participants in the 12 RDS online surveys were men whose gender at birth was male, were at least 18 years old, residing for at least 3 months in the province where the RDS online survey was being conducted, and who have had anal or oral sex with another man in the last year or do not prefer sex with women only. Regarding the last 2 criteria, the participants were first asked if they had anal or oral sex with another man in the last year and only those who responded *no* were subsequently asked if they prefer sex with women only. To be eligible, participants had to answer *yes* to the former question about having had sex with another man, or if they answered *no* to that question, they would have had to answer *no* to the subsequent question about preferring sex with women only.

The following study procedures were followed for the RDS online survey. Between 6 and 8 seed individuals were identified in each province and given 3 coupons each to recruit their peers to respond to the RDS online survey, who would in turn be given 3 coupons each, and so on. Recruits who did not have access to the internet to complete the RDS online survey or preferred to provide answers offline were provided with a telephone number to contact a member of the investigation team who would collect information from them over the phone or in person and enter it online.

### Estimation of Population Size

The nonidentifying information collected from the 12 RDS online surveys was analyzed to calculate the proportion of respondents who answered *yes* to having used Jack’d in the past month with a 95% CI. In each province, 2 different estimators, Gile’s sequential sampling (Gile’s SS) and Salganik-Heckathorn (RDS-I), were used to generate estimates for the survey respondents’ Jack’d social app use [15,16].

Homophily tests were conducted to assess the extent of random referrals from respondents to their personal networks [17]. A homophily test value less than 1 indicates respondents with similar characteristics in successive waves of peer recruitment. A test of the sensitivity of the estimator was conducted on the proportion of survey participants who answered *yes* to having used Jack’d in the past month. When the predicted proportion fell within the range of 0.2 to 0.8, we considered the estimator to be not sensitive [18]. Additional tests were conducted on the provincial data to determine if there are significant social, behavioral, and demographic differences between participants who had used Jack’d and those who had not.

Convergence plots in RDS were 1 indicator of having sufficient data collected to get a reliable estimate. When the key estimator remained stable within 2% of the sample proportion, we predicted that additional responses collected would yield insignificant changes to the estimate [19]. Bottleneck plots were also created to show the differences between the individual seeds in each province and determine if they had converged.

The RDS-I estimator tends to underestimate the result if the sampling does not converge, and the Gile’s SS bootstrap tends to underestimate the result if the homophily test value is less than 1 or there is bias in seed selection [20]. On the basis of the convergence and homophily tests, the results of 1 estimator were selected to estimate the population size in each province. The number of Jack’d users counted in each province over 1 month was divided by the selected estimator results of the proportion of RDS survey respondents who said *yes* to having used Jack’d in the past month in that province, to arrive at the provincial MSM population size estimate.

The population size estimates were converted to a percentage of the general adult male population (15-49 years) and compared with the range of percentages reported in the 2018 Spectrum Quick Start Guide [21,22]. The estimates of 7 provinces in this study were extrapolated to 50 other provinces of Vietnam according to the geographic and socioeconomic regional grouping of provinces [23-25]. The percentage of MSM among the adult male population, weighted based on proportions of Jack’d users within each age group (15-19, 20-24, 25-29, 30-34, 35-39, 40-44, and 45-49 years), was applied to the matching provinces. The sum of 11 directly observed and 50 extrapolated provincial estimates in this study, along with 2 provincial estimates from previous piloting [9], provided the national MSM population size. Steps of the extrapolation process and calculations are detailed in Multimedia Appendix 1.

Ethical approval for this study was obtained from the Institutional Review Board of the Hanoi School of Public Health (Approval no. 298/2016/YTCC-HD3, IORG no. 0003239; FWA number: 0009326). The protocol was also reviewed and approved by the US Centers for Disease Control and Prevention human subjects research office.

## Results

Hanoi had the highest number of active Jack’d users among the 12 provinces at 12,848 persons, and Binh Dinh province had the lowest number of active users at 260 persons. The median age of Jack’d users in the provinces was 25 years, with 75% of the users in the 19 to 35 years age group. The median age of the RDS survey respondents was 24 years, with 75% of the users in the 19 to 32 years age group. There was no statistically significant difference between the mean age or the mean network size of respondents who answered *yes* to having used Jack’d in the past month and those who answered *no* (*t*_22_=1.381; *P*=.18 and *t*_21_=1.055; *P*=.304, respectively). There was a significant difference between users of Jack’d and nonusers of Jack’d having had sex with another man in the last year (*χ*^*2*^_*1*_=41.0; *P*<.001). The numbers of participants at each stage of the RDS survey, their social and demographic characteristics, and the summary measures of Jack’d use are presented by province in Table 1. Among the 2177 eligible respondents, 5% completed the survey by telephone and the rest completed the survey online.

**Table 1:** Number of respondent-driven sampling survey participants and respondents, their social and demographic characteristics, and summary measures of their Jack’d usage.

Provinces	Participants		Characteristics of participants			Summary measures	
	Survey participants, n	Eligible survey respondents, n	Mean age (years)	Mean network size	Participants who did not have sex with another man in the last 12 months but who did not prefer sex with women only among eligible survey respondents, n	Participants with a Jack’d account, n	Participants using Jack’d in the last 30 days, n
An Giang	173	167	22	8	39	33	18
Bac Giang	132	125	28	46	28	77	52
Binh Dinh	134	126	27	5	26	101	43
Can Tho	195	167	23	6	28	37	19
Da Nang	193	167	24	5	17	114	80
Dak Lak	123	121	24	8	52	41	21
Dong Nai	238	227	25	7	15	69	17
Dong Thap	217	212	24	9	61	47	33
Hanoi	296	264	23	13	22	209	130
Hai Phong	345	279	25	10	70	155	96
Nam Dinh	132	122	25	10	21	84	65
Thanh Hoa	244	200	25	9	34	107	67
Total	2422	2177	24.3	10.6	413	1074	641

Among the 12 provinces, 4 (Dak Lak, Dong Thap, An Giang, and Can Tho) had sensitivity ratios that were outside the 0.2 to 0.8 range when using Jack’d use in the last 30 days as the estimator. Among the 12 provinces, 2 (Dong Nai and An Giang) had sensitivity ratios that were outside the 0.2 to 0.8 range when using all-time Jack’d usage as the estimator. Among the 12 provinces, 4 (Bac Giang, Can Tho, Dak Lak, and Dong Nai) had homophily values less than 1 when using Jack’d use in the last 30 days as the estimator and 1 (Nam Dinh) had homophily value less than 1 when using all-time Jack’d use as the estimator. The convergence plots in 10 provinces showed that the RDS survey samples converged; however, in 2 provinces, Dak Lak and Thanh Hoa, there was bottlenecking between seeds (see Multimedia Appendix 2). As there were more provinces with a homophily value less than 1 than provinces whose RDS survey sample did not converge, we used the RDS-I estimator, which may be less prone to underestimate the result. An Giang was the only province with its sensitivity ratio outside of the 0.2 to 0.8 range for both 30-day use of Jack’d and all-time use of Jack’d. It is also the province with a sensitivity ratio furthest outside of the 0.2 to 0.8 range. For these reasons, it was considered that the RDS estimator failed to produce a reliable estimate for An Giang.

Analysis of the data shows that on average 37.5% of MSM in the 11 provinces with reliable estimates had used Jack’d in the last 30 days (95% CI 27.0-47.9). Among these provinces, Can Tho had the lowest percentage of active Jack’d users in the last 30 days at 11.4% (95% CI 3.3-19.4) and Da Nang had the highest percentage at 42.9% (95% CI 28.3-57.6). The average weighted percentage of MSM ever using Jack’d in the 11 provinces was 56.7% (95% CI 47.8-65.5). In 1 province, Dong Nai, the 30-day RDS-I and Gile’s SS estimates did not produce statistically meaningful results, and the all-time Jack’d use estimator was used instead in this province.

The highest population size of MSM aged 18 to 49 years among the 11 provinces with reliable estimates was in Hanoi at 30,417 persons (95% CI 24,656-39,691), and the lowest MSM population size was estimated in Binh Dinh at 743 persons (95% CI 559-1108). The average weighted percentage of MSM among males aged 15 to 49 years in the 11 provinces was 0.96%, with a range of percentages from 0.70% to 2.47%. The complete results are presented in Table 2.

**Table 2:** Estimated number of men who have sex with men aged 18 to 49 years and weighted percentage of men who have sex with men among males aged 15 to 49 years in 11 provinces of Vietnam.

Province	Participants counted on Jack’d over 30 days, n	Proportion of MSM^a^ using Jack’d (last 30 days, RDS-I^b^)	95% CI for proportion of MSM using Jack’d (last 30 days, RDS-I)	Estimated population size of MSM aged 18-49 years	95% CI for estimated population size of MSM aged 18-49 years	Weighted percentage of MSM among males aged 15-49 years
Bac Giang	360	0.42	0.28-0.55	864	653-1274	0.23
Binh Dinh	260	0.35	0.23-0.47	743	559-1108	0.21
Can Tho	713	0.11	0.03-0.19	6276	3677-21,418	1.22
Da Nang	1713	0.43	0.28-0.58	3990	2974-6059	1.70
Dak Lak	279	0.15	0.07-0.23	1895	1226-4180	0.46
Dong Nai	1191^c^	0.15^d^	0.02-0.28	7759	4216-68,370	1.10
Dong Thap	303	0.14	0.06-0.21	2181	1420-4711	0.48
Hanoi	12,848^e^	0.42	0.32-0.52	30,417	24,656-39,691	1.81
Hai Phong	1141	0.34	0.25-0.43	3336	2645-4515	0.73
Nam Dinh	446	0.39	0.26-0.52	1131	850-1687	0.29
Thanh Hoa	670^e^	0.22	0.11-0.33	3017	2032-5846	0.40
Total	19,924	0.37	0.27-0.48	61,609	44,909-158,860	0.96

^a^MSM: men who have sex with men.

^b^RDS: respondent-driven sampling.

^c^Count is for all-time use of Jack’d using the capture-recapture method.

^d^Estimate is for all-time use of Jack’d (RDS-I).

^e^Count obtained directly from social app service provider.

Extrapolation of the results from the 11 provinces with reliable estimates to the national MSM population size resulted in an estimate of 178,000 MSM (95% CI 122,000-512,000) in Vietnam. The percentage of MSM among adult males aged 15 to 49 years in Vietnam is 0.68% (95% CI 0.46-1.95).

## Discussion

### Principal Findings

Our estimates are the first comprehensive national estimation of the MSM population size conducted in Vietnam that use an empirical method. The point estimate of the 15- to 49-year-old MSM population in Vietnam produced in this study is 178,000, with an estimated range from 122,000 to 512,000. The corresponding estimated percentage of MSM among adult males aged 15 to 49 years of 0.68% is within the range of 0.09% to 4.06% suggested for the Asia and Pacific region by the Joint United Nations Programme on HIV/AIDS (UNAIDS) Spectrum guideline; however, the guideline does not provide a functional definition of MSM, which limits the comparability of the results [21]. The estimated percentage of MSM among adult males aged 15 to 49 years in Vietnam also agrees with recent estimations in other low- and middle-income countries (eg, [26-30]).

The specific definition of MSM adopted in this study based on behavior and sexual preference has broader inclusion criteria than internet-based surveys, which only include men who have been sexually active with a man in the past year in their analysis [31,32]. This study is also different from surveys that measure population sizes based on sexual orientation or gender identity. For example, the 2014 US National Health Statistics Report uses self-identification methods to estimate that 1.6% of adults in the United States are gay or lesbian [33]. Public health researchers prefer the behavioral and temporal definition of MSM over identities because behaviors, not identities, lead to sexual transmission risk [34]. Self-identification of sexual orientation can also be biased by social stigma, either underestimating or overestimating nonheterosexual population sizes [35-37]. The use of an anonymous RDS online survey propagated through social networks of the MSM community reduces the effect of social stigma in our study results.

The provincial estimates in this study were lower in Bac Giang, Binh Dinh, Dak Lak, Dong Thap, Nam Dinh, and Thanh Hoa; within the range in Da Nang, Dong Nai, Hanoi, and Hai Phong; and higher in Can Tho, in comparison with the expected range of MSM population sizes reported in past HIV and AIDS estimates and projection reports [2,38]. The provinces in this study were selected to be representative of other unsampled provinces in the country with similar geographic and socioeconomic characteristics [23-25]. During extrapolation, the age-disaggregated population of unsampled provinces was used to weight the population proportion of MSM among adult males. There may be other factors that influence the proportional population of MSM in the provinces for building the strata for the extrapolation or for weighting of proportions. For instance, there are different levels of social stigma experienced by key populations at risk of HIV in different geographic areas, and this may be a factor in variations in MSM population size in the provinces [39]. In consideration of variability across provincial estimates, additional studies are required for future extrapolations of MSM population size and further generation of stigma and discrimination data at the provincial level.

According to our estimates, during a given month, nearly 2 out of 3 people with a Jack’d account are actively connecting with other MSM on the social app, and there is a significant relationship between use of the social app and having sex with other men. These results are consistent with other recent studies that indicate increasing use of online social networks by MSM to find partners, while bypassing social stigma [40,41]. These results also imply the potential for rapid, targeted, and cost-effective outreach to the MSM community through the social apps with messaging on health and social services available in their vicinity. Future analysis may look at the relationship between social app usage and sexual risk behavior. Further studies will also elucidate if members of the MSM community who are on social apps are socially well connected to other MSM or not, which will have important implications for the expected yield of different communication campaigns.

Overall, 3 different approaches were used to obtain the count of Jack’d users for the multiplier method in this study. Each of these approaches comes with its own advantages and disadvantages. The direct counting of users on social apps is a resource-intensive process. It requires investigators to manually record characteristics of active users on the social app daily or more frequently to not miss any peak periods when users log into the apps. In peak periods and in large cities with thousands of users, this process may not be feasible. Moreover, this approach also requires the deduplication of users in the records, which is also a resource-intensive step in the process. However, the advantage of this method is that the resulting number of active users during a brief period of 1 month reduces the recall bias when survey respondents in the second part of the multiplier method are asked about their use of the app in the past month.

The second approach to obtain the count of Jack’d users for the social app multiplier method in this study was obtaining the data directly from the app service provider. The advantage of this approach is that it requires little time, there is no need for deduplication, there is higher accuracy in the count than the manual count, and there is greater privacy as 1 integer figure is collected as the sum of all active users. However, these advantages are traded off with the cost of purchasing the aggregate, unduplicated, and nonidentifiable number of active users from the service provider. The third approach to obtain the count of users was a capture-recapture method on Jack’d. This approach required only 2 counts on 2 distinct days on Jack’d and did not require any deduplication. The disadvantage of this method is that it produces a count of all active users at any time on the social app, which in turn requires a less precise question in the RDS survey that is prone to recall bias. Future research should consider the reliability and precision of the data generated by these approaches as additional criteria to decide on the approach for use with the social app multiplier method.

### Limitations

The multiplier method requires the independence of the 2 data sources, the population in the 2 data sources to be defined the same way, and the 2 data sources to have aligned time periods and geographic areas [13]. This study attempts to address these requirements of the multiplier method. For example, we limit the time between the count of users on Jack’d and the RDS online survey. Furthermore, the 2 data sources match in the 1-month timeframe of the data collection and within each of the 12 provinces where data are collected. In terms of population definition, an assumption is made that the sexual behavior or preference of the Jack’d users matches the inclusion criteria of the participants in the RDS online survey. In matching the age of participants in the 2 data sources, the RDS survey participants are asked about their age, and only those reporting to be at least 18 years old are eligible to participate, similar to the Jack’d requirement of being at least 18 years old to download and use the app. However, as verifying ages on social apps and online surveys is enormously complicated and contentious, and considering literature that reports up to one-fifth of dating profiles and survey respondents being inaccurate in reporting their age [42-44], there may be some skewing of the results in this study because of misreporting of age. Future research should validate the assumptions of the multiplier method and other assumptions of the RDS data by inviting a sample of social app users and RDS survey respondents to a follow-up survey based on standard RDS diagnostic questions [45], along with questions on the participants’ age and their sexual behavior and preferences.

Although the age profile of Jack’d users and RDS survey respondents in the 12 provinces included in this study was comparable, these age profiles are skewed toward a younger age range than Vietnam’s total male population pyramid [46]. This youth bulge and underrepresentation of older MSM on social media are not uncommon, and studies of the MSM population in Europe and South America speculate that lower internet literacy and increasing proportions of older MSM living in settled relationships may be among factors related to the skewed demographics [47,48]. As noted earlier on misreporting of age, it is plausible that in Vietnam there may also be some MSM younger than 18 years reporting an older age to download and use the Jack’d app and older MSM reporting a younger age on their profile. Acknowledging the low proportion of older participants and some bias in our dataset toward the younger population of MSM, we limit the generalization of our results to the maximum age of 49 years; however, we do not have sufficient data on the extent and amplitude of skewing to attempt any corrective adjustments to the age demographic information.

Intraprovince migration of MSM risks some bias in the population size estimates as the RDS online survey excludes individuals who report having moved within the past 3 months even though there is no way to be sure if the Jack’d users had moved to the province in the past 3 months. We attempt to reduce the possibility of counting short-term migrants and visitors by only counting users who appear at least twice on the social app at 2 distinct days spaced over 1 month.

### Conclusions

The estimation of MSM in Vietnam reported in this study is within the bounds suggested by UNAIDS for countries in the Asia and Pacific region, and the range produced in this study comfortably includes the estimated number of MSM in Vietnam arrived at through the national technical working group profiling and modeling process. The current estimation is based on an empirical method that relies on well-known and tested techniques along with innovative use of social apps used by the MSM population in Vietnam. This study highlights the feasibility of reaching a large percentage of MSM through a social app with programmatic and health promotion interventions. It is also the first time that population size estimations have been conducted in the provinces included in this study, and where reliable estimates were obtained, this study provides those provinces with valuable information on MSM population sizes that they can begin to work with in program planning and resource allocation. In other provinces where the population size estimates were extrapolated to but not directly observed, this study recommends that the extrapolated estimates be validated using locally appropriate, empirical size estimation methods, including the reliable methods and technologies that were introduced in this study. In provinces where there was a degree of homophily, bottlenecking, and sensitivity in the RDS survey results or where the estimators failed to produce reliable results, alternative methods should be attempted to assess and validate the MSM population sizes. Although the national estimation in this study gets closer to defining the potential range of the number of MSM in Vietnam, future studies will be needed to validate the range and further specify the estimated number. As the MSM population size is one of the key inputs to the national AIDS epidemic modeling and projection process, the AIDS epidemic model needs to be reviewed and updated with the new estimation.

## Appendix

Multimedia Appendix 1 Method of extrapolation from the study provinces and calculation of the national population size of men who have sex with men.

Multimedia Appendix 2 Convergence, bottlenecking, sensitivity, and homophily in the respondent-driven sampling survey.

## Abbreviations

Gile’s SS: Gile’s sequential sampling
MSM: men who have sex with men
RDS: respondent-driven sampling
UNAIDS: Joint United Nations Programme on HIV/AIDS
