# Supplementary material for: Estimation of the Population Size of Men Who Have Sex With Men in Vietnam: Social App Multiplier Method
Source: JMIR Public Health Surveill. 2019 Apr 17;5(2):e12451. doi: 10.2196/12451 (PMC6492067; doi:10.2196/12451)
Supplement: Multimedia Appendix 1 [file publichealth_v5i2e12451_app1.pdf]

Method of Extrapolation from the study provinces and calculation of the national population size of MSM:

Step 1: Calculate population of MSM 15 to 49 years old in the reference province that produced reliable estimates, weighted by the proportion of Jack'd users in each age group:

$$P_{ry[15\text{-}49]} = p_{ry[18\text{-}49]} * (n_{ry[15\text{-}19]} * q_{18\text{-}19} + n_{ry[20\text{-}24]} * q_{20\text{-}24} + n_{ry[25\text{-}29]} * q_{25\text{-}20} + n_{ry[30\text{-}34]} * q_{30\text{-}34} + n_{ry[35\text{-}39]} * q_{35\text{-}39} + n_{ry[40\text{-}44]} * q_{40\text{-}44} + n_{ry[45\text{-}49]} * q_{45\text{-}49}) * 7 \;/\; n_{ry[18\text{-}49]}$$

Where $p_{ry[18\text{-}49]}$ is the estimated size of MSM population (18-49) in the reference province y obtained by the multiplier method in this study, $q_{i\text{-}j}$ is the percentage of national Jack'd users in each age group i-j, and $n_{r\ i\text{-}j}$ is the total male population in each age group i-j of the reference province y.

Note: The Viet Nam general population data is available in 5-year increments, including the 15-19 age group. Due to the limitation of working with males 18 and older in this study, to calculate the needed 18-19 age group in the population an assumption was made that $n_{ry[18\text{-}19]} = n_{ry[15\text{-}19]}*2/5$

Step 2: Extrapolate the MSM population (15-49) from the reference province to another province x in the reference region:

$$p'_{x[15\text{-}49]} = P_{ry[15\text{-}49]} * (n_{x[15\text{-}19]} * q_{18\text{-}19} + n_{x[20\text{-}24]} * q_{20\text{-}24} + n_{x[25\text{-}29]} * q_{25\text{-}20} + n_{x[30\text{-}34]} * q_{30\text{-}34} + n_{x[35\text{-}39]} * q_{35\text{-}39} + n_{x[40\text{-}44]} * q_{40\text{-}44} + n_{x[45\text{-}49]} * q_{45\text{-}49}) * 7 \;/\; n_{ry[15\text{-}49]}$$

Where $n_{x[i\text{-}j]}$ is the total male population in each age group i-j of the extrapolated province x. Step 2 is repeated for each province linked to a reference province listed under the "Extrapolated from Province" in the Table below.

Step 3: Calculate national population size of MSM 15-49:

$$p_{MSM15\text{-}49} = p'_{1\,[15\text{-}49]} + p'_{2\,[15\text{-}49]} + \ldots + p'_{50\,[15\text{-}49]} + p_{r1\,[15\text{-}49]} + p_{r2\,[15\text{-}49]} + \ldots + p_{r11\,[15\text{-}49]} + p_{hcmc} + p_{na}$$

Where $p'_{x\,[15\text{-}49]}$ are the MSM population (15-49) in the 50 provinces where estimates were extrapolated to, $p_{ry\,[15\text{-}49]}$ are the MSM population (15-49) in the 11 reference provinces where reliable estimates were directly obtained in this study, and p_{hcmc} and p_{na} are the MSM population (15-49) in Ho Chi Minh City and Nghe An province respectively which were previously estimated during a piloting of this method in 2016.

Region	Province	Extrapolated from province
Central Highlands	Dak Lak	
Central Highlands	Dak Nong	Dak Lak
Central Highlands	Gia Lai	Dak Lak
Central Highlands	Kon Tum	Dak Lak
Central Highlands	Lam Dong	Dak Lak
Mekong Delta	An Giang	Dong Thap
Mekong Delta	Bac Lieu	Dong Thap
Mekong Delta	Ben Tre	Dong Thap
Mekong Delta	Ca Mau	Dong Thap
Mekong Delta	Can Tho	
Mekong Delta	Dong Thap	
Mekong Delta	Hau Giang	Dong Thap
Mekong Delta	Kien Giang	Dong Thap
Mekong Delta	Long An	Dong Thap
Mekong Delta	Soc Trang	Dong Thap
Mekong Delta	Tien Giang	Dong Thap
Mekong Delta	Tra Vinh	Dong Thap
Mekong Delta	Vinh Long	Dong Thap
North Central Coast	Ha Tinh	Thanh Hoa
North Central Coast	Nghe An	
North Central Coast	Quang Binh	Thanh Hoa
North Central Coast	Quang Tri	Thanh Hoa
North Central Coast	Thanh Hoa	
North Central Coast	Thua Thien Hue	Thanh Hoa
Northeast	Bac Giang	
Northeast	Bac Kan	Bac Giang
Northeast	Cao Bang	Bac Giang
Northeast	Ha Giang	Bac Giang
Northeast	Lang Son	Bac Giang
Northeast	Phu Tho	Bac Giang
Northeast	Quang Ninh	Bac Giang
Northeast	Thai Nguyen	Bac Giang
Northeast	Tuyen Quang	Bac Giang
Northwest	Dien Bien	Bac Giang
Northwest	Hoa Binh	Bac Giang
Northwest	Lai Chau	Bac Giang
Northwest	Lao Cai	Bac Giang
Northwest	Son La	Bac Giang
Northwest	Yen Bai	Bac Giang
Red River Delta	Bac Ninh	Nam Dinh
Red River Delta	Ha Nam	Nam Dinh
Red River Delta	Ha Noi	
Red River Delta	Hai Duong	Nam Dinh
Red River Delta	Hai Phong	
Red River Delta	Hung Yen	Nam Dinh
Red River Delta	Nam Dinh	
Red River Delta	Ninh Binh	Nam Dinh
Red River Delta	Thai Binh	Nam Dinh

Red River Delta	Vinh Phuc	Nam Dinh
South Central Coast	Binh Dinh	
South Central Coast	Binh Thuan	Binh Dinh
South Central Coast	Da Nang	
South Central Coast	Khanh Hoa	Binh Dinh
South Central Coast	Ninh Thuan	Binh Dinh
South Central Coast	Phu Yen	Binh Dinh
South Central Coast	Quang Nam	Binh Dinh
South Central Coast	Quang Ngai	Binh Dinh
Southeast	B.Ria-V.Tau	Dong Nai
Southeast	Binh Duong	Dong Nai
Southeast	Binh Phuoc	Dong Nai
Southeast	Dong Nai	
Southeast	Ho Chi Minh City	
Southeast	Tay Ninh	Dong Nai
