# Supplementary material for: Estimation of the Population Size of Men Who Have Sex With Men in Vietnam: Social App Multiplier Method
Source: JMIR Public Health Surveill. 2019 Apr 17;5(2):e12451. doi: 10.2196/12451 (PMC6492067; doi:10.2196/12451)
Supplement: Multimedia Appendix 2 [file publichealth_v5i2e12451_app2.pdf]

Province	Convergence (on Jack'd use in last 30 days)	Convergence (on all time Jack'd usage)	Bottlenecking (on Jack'd use in last 30 days)	Bottlenecking (on all time Jack'd usage)
**An Giang**	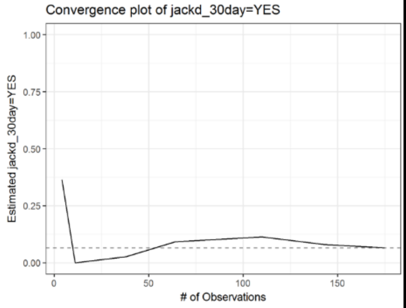	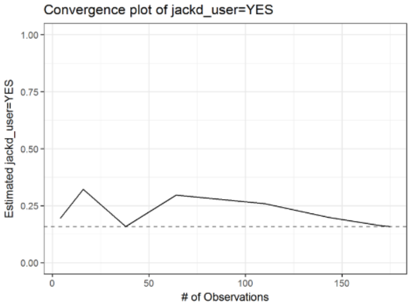	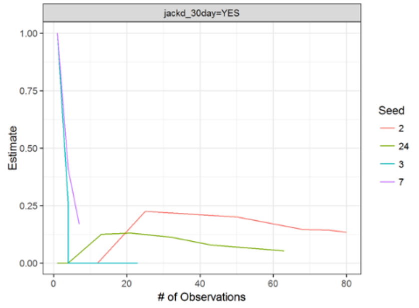	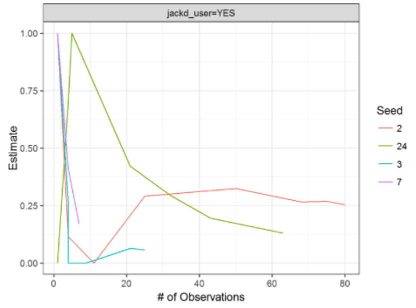
**Bac Giang**	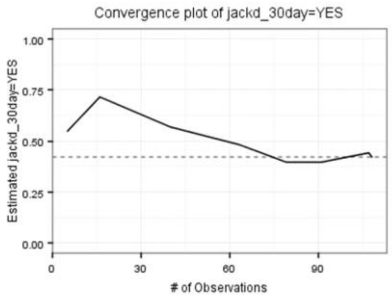	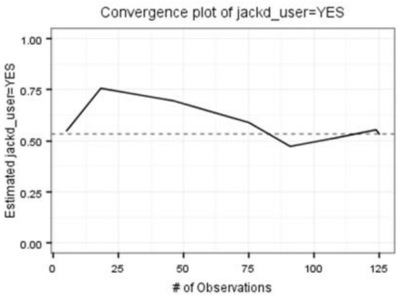	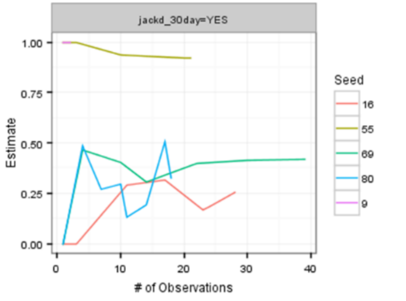	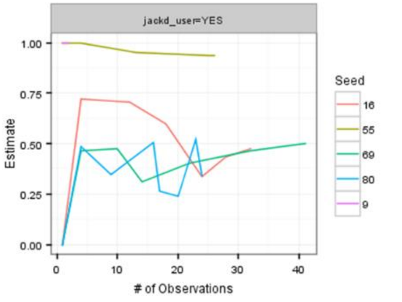
**Binh Dinh**	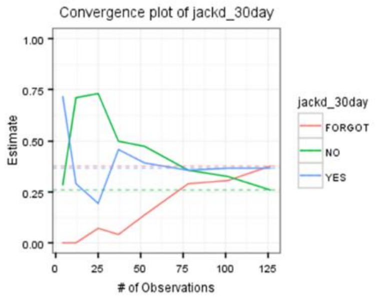	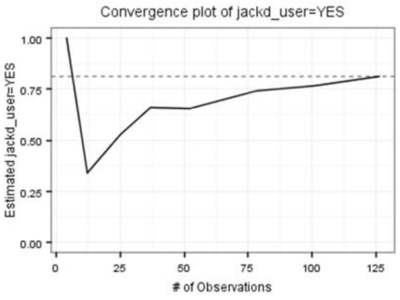	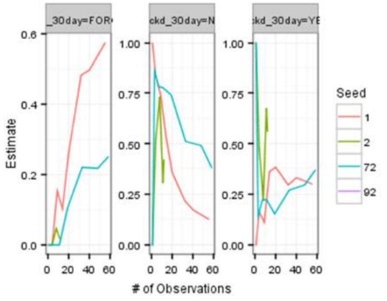	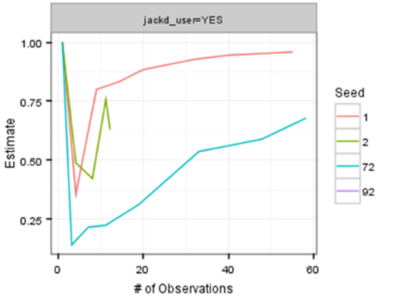

Can Tho

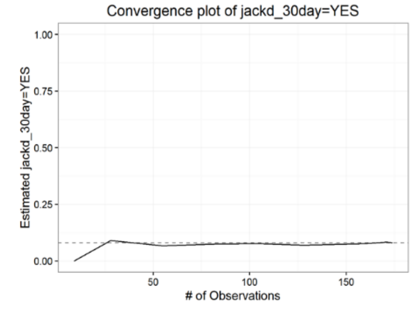

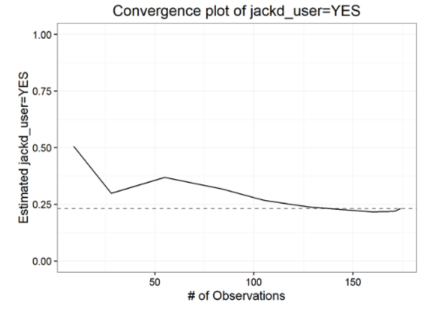

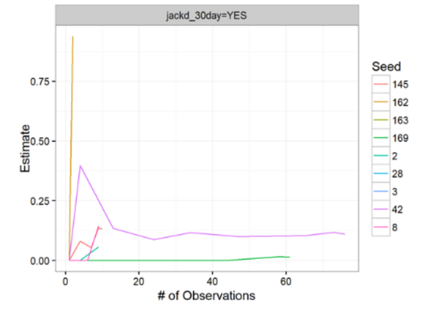

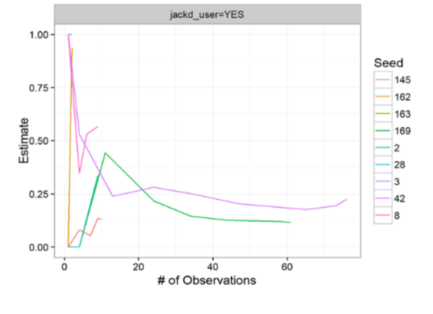

Da Nang

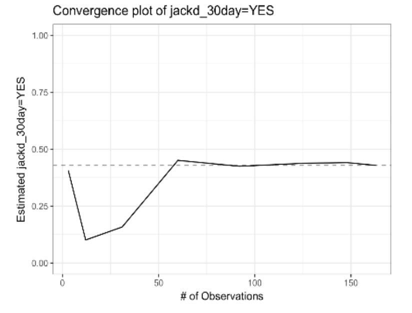

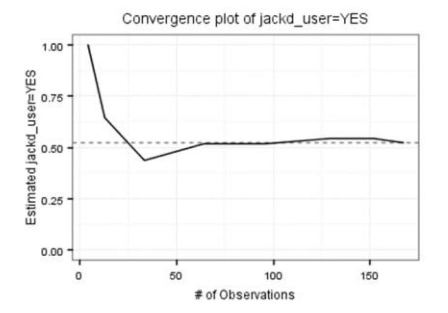

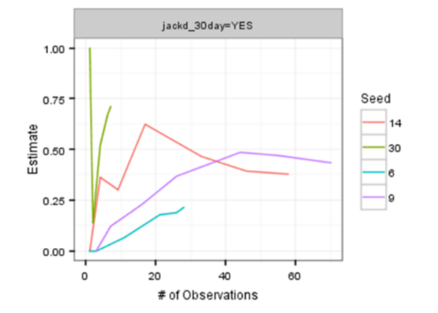

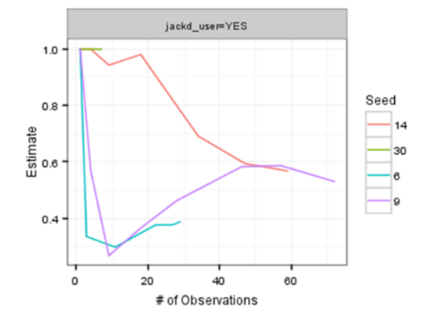

Dak Lak

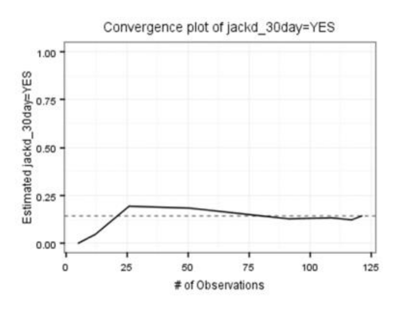

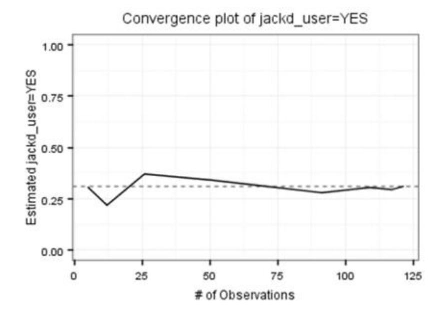

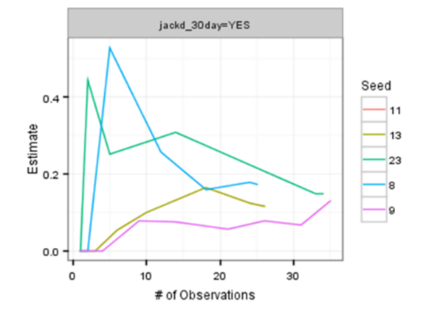

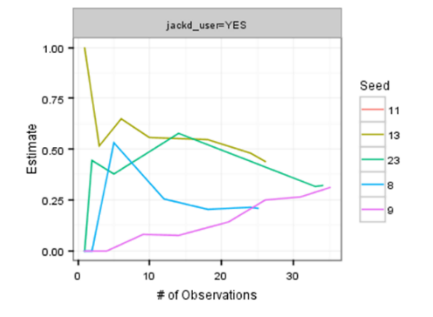

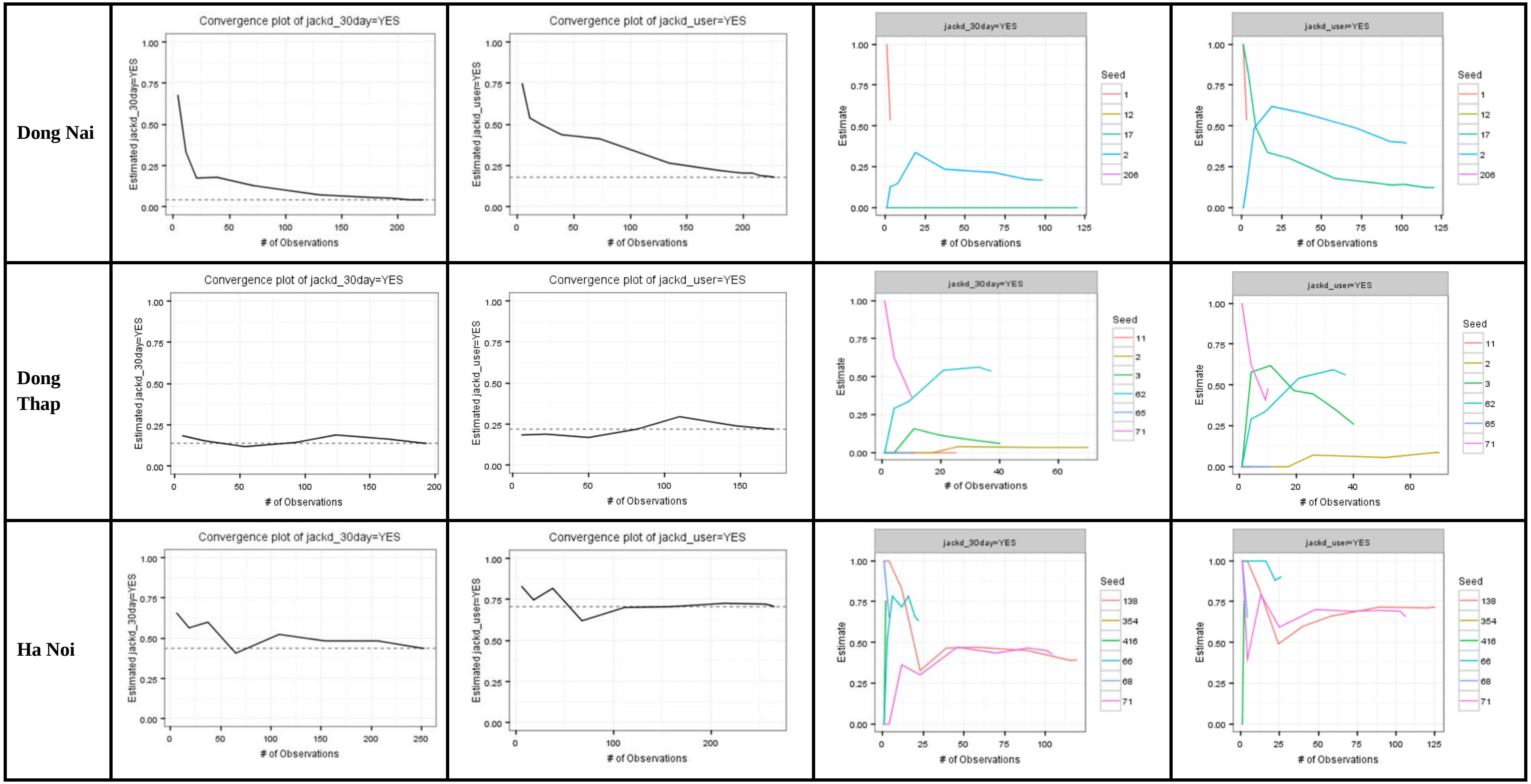

Dong Nai
Dong Thap
Ha Noi
Convergence plot of jackd_30day=YES
Convergence plot of jackd_user=YES
Estimated jackd_30day=YES
Estimated jackd_user=YES
# of Observations
jackd_30day=YES
jackd_user=YES
Estimate
Seed

<table>
<tr>
<td>Hai Phong</td>
<td>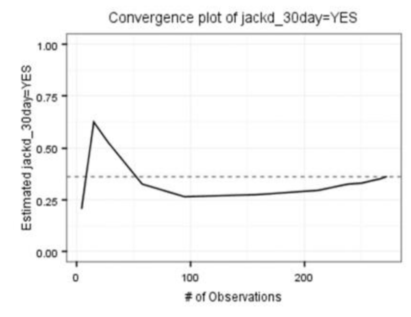
</td>
<td>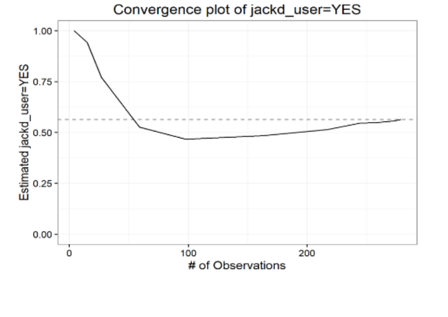
</td>
<td>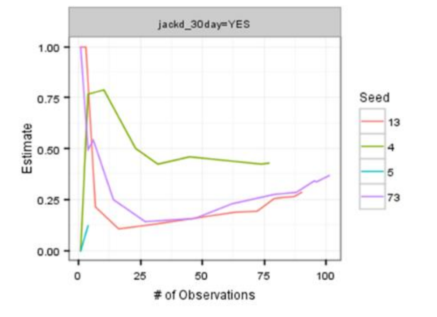
</td>
<td>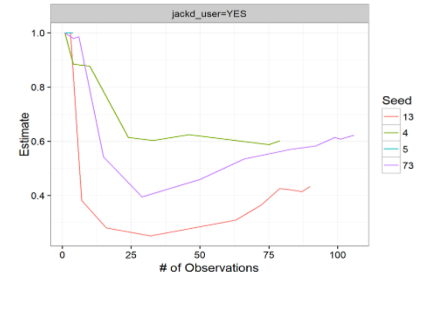
</td>
</tr>
<tr>
<td>Nam Dinh</td>
<td>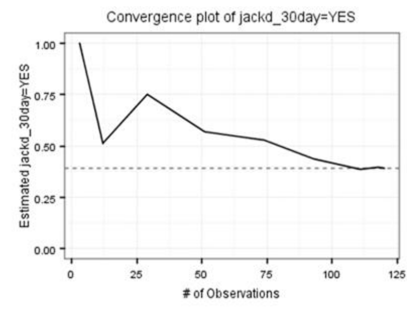
</td>
<td>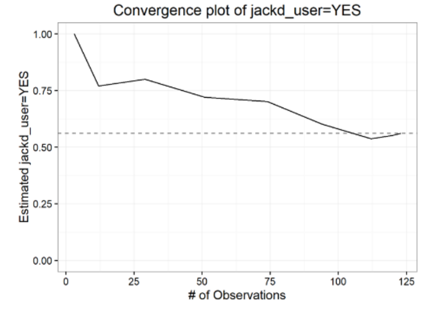
</td>
<td>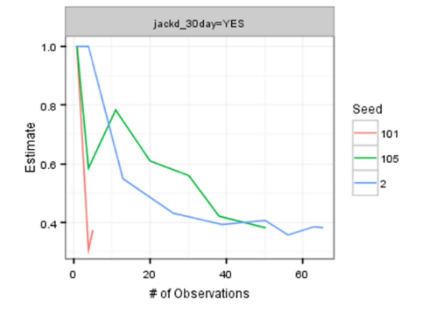
</td>
<td>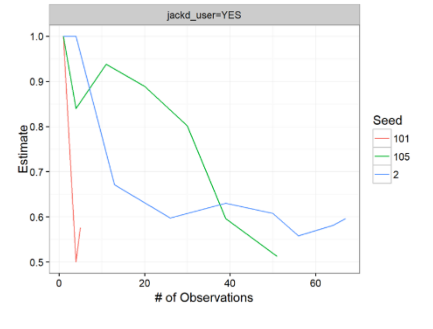
</td>
</tr>
<tr>
<td>Thanh Hoa</td>
<td>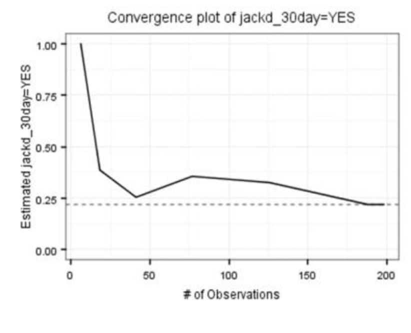
</td>
<td>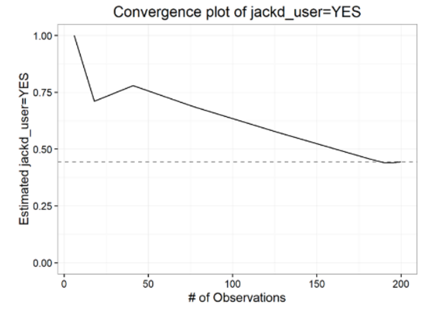
</td>
<td>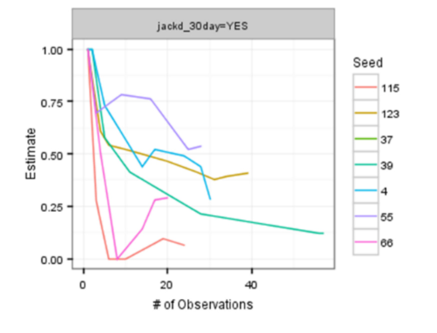
</td>
<td>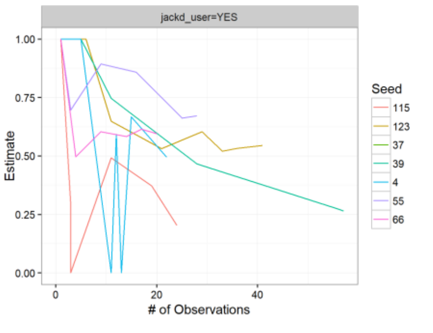
</td>
</tr>
</table>

Province	Sensitivity (on Jack’d use in last 30 days) (%)	Sensitivity (on all time Jack’d usage) (%)	Homophily (on Jack’d use in last 30 days)	Homophily (on all time Jack’d usage)
**An Giang**	10.7	19.5	1.51	1.25
**Bac Giang**	41.7	60.6	0.61	1.04
**Binh Dinh**	32.1	75.4	1.35	1.76
**Can Tho**	11.3	24.7	0.94	2.15
**Da Nang**	41.6	59.9	1.79	1.52
**Dak Lak**	17.1	33.3	0.99	1.12
**Dong Nai**	29.9	7.1	0.99	1.77
**Dong Thap**	15.6	22.2	1.53	1.59
**Ha Noi**	59.5	78.1	1.46	1.91
**Hai Phong**	27.8	44.9	1.25	1.13
**Nam Dinh**	49.2	63.6	1.00	0.92
**Thanh Hoa**	33.8	43.7	1.36	1.26
